# MRI of Neurogenic Human Motor Units Following Poliomyelitis

**DOI:** 10.1002/mus.70107

**Published:** 2025-12-14

**Authors:** Stuart Maitland, Matthew Birkbeck, Ian Schofield, Lawrence Best, James Scott, Andrew Blamire, Roger G. Whittaker

**Affiliations:** ^1^ Translational and Clinical Research Institute, Newcastle University Newcastle upon Tyne UK; ^2^ Northern Medical Physics and Clinical Engineering Freeman Hospital, Newcastle upon Tyne NHS Foundation Trust Newcastle upon Tyne UK; ^3^ Ageing, Sarcopenia and Multimorbidity Theme, NIHR Newcastle Biomedical Research Centre, Newcastle upon Tyne Hospitals NHS Foundation Trust, Cumbria Northumberland Tyne and Wear NHS Foundation Trust and Faculty of Medical Sciences Newcastle University Newcastle upon Tyne UK

**Keywords:** electromyography, magnetic resonance imaging, motor unit, poliomyelitis, reinnervation

## Abstract

**Introduction/Aims:**

Surviving motor units in neurogenic diseases demonstrate collateral reinnervation. Scanning electromyography (EMG) reveals normal motor unit corridor length, but with “silent regions,” suggesting that reinnervation does not result in increased motor unit size but may increase motor unit complexity. Motor unit magnetic resonance imaging (MUMRI) pairs MR imaging with electrical nerve stimulation to visualize individual motor units. This study aimed to assess the motor unit dimensions and complexity in patients with previous poliomyelitis compared to healthy controls.

**Methods:**

Patients with a history of polio were recruited from the British Polio Fellowship, compared to a retrospective cohort of healthy controls. They underwent medical history and examination of lower limb power, fatigue assessment (fatigue severity score, FSS), and a 3 T MUMRI scan of the less‐affected lower limb. The cross‐sectional area, maximum, and minimum Feret diameter of the motor unit territories in tibialis anterior were calculated. Motor unit complexity was computed using the Hausdorff box‐counting method.

**Results:**

Of 12 polio survivors, *n* = 8 (6 female) were suitable for analysis and were compared to 19 controls. The mean motor unit maximum Feret diameter was 10.3 ± 3.1 mm compared to 8.4 ± 5.2 mm in controls (*p* = 0.34). The mean shape complexity was 0.59 ± 0.12 compared to 0.45 ± 0.2 in controls (*p* = 0.03).

**Discussion:**

Polio survivors demonstrate motor units with normal dimensions but increased shape complexity, indicating nonuniform collateral reinnervation largely limited to existing territories. The size and shape of motor units could help in understanding the physiological processes behind reinnervation, both in polio and other neurogenic diseases such as amyotrophic lateral sclerosis.

## 1 Introduction

Understanding the neurogenic remodeling process is key to understanding the changes seen in altered motor unit potential (MUP) morphology in neurogenic diseases that form the basis of clinical electromyography (EMG) interpretation. However, it remains unclear whether reinnervation increases the spatial territory of a motor unit, or if it occurs exclusively within the original boundary.

While concentric EMG shows neurogenic MUPs are characterized by increased amplitude and duration, this is largely a product of the closest ~10 fibers [1] and reflects increased fiber density in the 1 mm around the needle [1] rather than the whole 10–20 mm motor unit territory [2, 3, 4].

While macro‐MUP amplitude increases in neurogenic diseases [5, 6, 7, 8] indicating an increased total fiber count for the whole motor unit, and single‐fiber EMG confirms increased fiber density in polio survivors and other neurogenic conditions [9, 10], these methods do not measure spatial extent.

Attempts to measure territory directly have been contradictory. Scanning EMG has shown only minimal increases in motor unit dimensions in polio [11] or other neurogenic disorders [4], but with “silent regions” suggesting an increased complexity of the motor unit structure [12] whereas multielectrode EMG has shown significant increases in territory [13]. This variability may be explained in part by the random path of the electrode, which will tend to underestimate motor unit dimensions, especially those with complex profiles [14].

Animal studies using the glycogen depletion technique [15] have shown either a decrease in territory [15, 16, 17], a modest increase [18], or inconsistent results [19]. It therefore remains unclear whether the characteristic changes seen in neurogenic disease reflect an expanded motor unit territory or are solely due to increased fiber density.

We recently developed a magnetic resonance imaging (MRI)‐based technique (motor unit MRI or MUMRI) capable of imaging the size and shape of human motor units in vivo [20]. MUMRI uses a pulsed gradient spin echo (PGSE) sequence, which is inherently sensitive to the movement of water molecules. When combined with in‐scanner electrical nerve stimulation, the contraction of the muscle fibers in a given motor unit results in a localized signal void in the MR image. This in turn allows the quantification of the motor unit dimensions, cross‐sectional area, and shape [21]. The technique has been used to image single motor units in healthy controls [2], as well as to track the motor units through multiple axial slices of the lower leg, producing a 3D image of the motor unit [22].

In this study, we compared the motor unit dimensions of polio survivors who exhibit significant loss of motor units [23, 24, 25, 26], extensive motor unit re‐modeling [10] and very high amplitude MUPs [27], against a cohort of previously studied healthy controls. Our primary research question was whether or not compensatory reinnervation is limited to or extends beyond the normal territory of a human motor unit.

## 2 Methods

Ethical approval was obtained from the Newcastle University Faculty of Medical Sciences Ethics Committee (2603/34701). Participants were recruited via the British Polio Fellowship through local meetings and email newsletters.

Our inclusion criteria were participants with a self‐identified history of polio, defined as a febrile illness associated with acute muscle weakness followed by recovery with at least 15 years of stability [28]. Exclusion criteria were a contraindication to MRI scan including a pacemaker or implanted medical devices or metallic fragments, severe claustrophobia, and those with confounding neurological diagnoses. We did not exclude those with implanted orthopedic metalwork as some polio survivors have internal joint fixation to enable locomotion, and the scans could continue safely with these in place [29]. Participants were prescreened against inclusion and exclusion criteria via telephone and were invited to take part in the study. During the study visit, further medical history was obtained, and a neurological examination was performed of the lower limbs by an experienced consultant neurophysiologist to assess tone, power (using MRC grading 0–5), and degree and pattern of wasting. The fatigue severity score (FSS) questionnaire validated on patients with neurological disease [30] was completed by participants, consisting of agreement with nine statements on a scale from 1 to 7, giving a score from 9 to 63.

For comparison of motor unit sizes to healthy controls, we used historically acquired data from a study involving healthy older participants collected 2–3 years prior using the same protocol and scan system. For that study, ethical approval was obtained from Newcastle University Faculty of Medical Sciences Ethics Committee (1852/525/2020). All polio survivors and healthy controls gave written informed consent prior to participation.

### 2.1 Motor Unit MRI

The protocol for MUMRI has been described previously [2, 22]. Briefly, fibular nerve stimulation was paired with timed acquisition of PGSE images of the leg, with the slices centred at the thickest part of the calf. A 3 T Philips Achieva MR system (Best, the Netherlands) was combined with a flexible (FlexM, Philips) receiver coil pair positioned on either side of the leg. Several scans were performed (for detailed parameters see Table S1):

- T1‐weighted scan to assess muscle structure.
- Dixon sequences [31] to obtain the fat fraction in the lower legs bilaterally.
- MUMRI (PGSE) sequence setup scan during progressively increased stimulation strength through the range of muscle activation from subthreshold to supramaximal stimulation.
- MUMRI “single motor unit scan” using a narrower stimulation current range to target the activation of a single motor unit (alternation) as assessed by visual inspection during the scan. This was then repeated twice at an even narrower stimulation current range in two stacks of 12 slices each (no slice gap). The overlap between the stacks was two slices and, in each stack, the most distal slice was unstimulated, resulting in 21 stimulated slices, imaging a continuous volume extending 210 mm along the lower limb.

Poliomyelitis is an asymmetric, patchy process, and subclinical changes can be found even in less‐affected legs [32]. Based upon inspection of the structural T1‐weighted scan, we targeted for motor unit imaging the side that showed neurogenic change in the anterior compartment indicating involvement of poliomyelitis, but avoided a side if it had been completely replaced by fat leaving no muscle for investigation.

Peripheral nerve stimulation was performed using a pair of stimulating electrodes placed over the common fibular nerve at the fibular head. The electrodes were connected to a programmable stimulator (DS5; Digitimer, Ft. Lauderdale, Florida, USA) via coaxial cables with low pass filters (Minicircuits, New York, USA) at the Faraday cage. In all cases, the stimulator cathode was placed distal to the anode. The stimulus frequency was 1 Hz, with a bipolar square pulse of 0.3 ms duration. Stimulation current was progressively increased until a visible leg twitch was achieved. An initial MUMRI survey scan was then performed around this stimulation current in increments of 0.5 mA until a region of signal dropout was visible indicating the first recruitment of motor units, up to the point where the entire muscle image was seen to be saturated. This provided the current range required for initial motor unit recruitment through to stimulation of all available motor units. Detailed MU mapping scans were then conducted, focused on stimulation strengths where periods of alternation were seen during this setup scan. MUMRI scans were collected with 360 stimulated sequences, decrementing by increments of 0.03 mA over a total current range of 10.8 mA to identify single motor units on a single axial slice. To produce a map of the longitudinal extent of that motor unit, 125 stimulated sequences, decrementing by 0.03 mA (current range: 3.75 mA) were performed at 22 axial slices.

Motor units were identified based on alternation characteristics as described previously [2]. Regions of muscle were selected that exhibited “all or nothing” recruitment and did not have multiple levels of activation to suggest they were compounds of multiple motor units. The maximum and minimum Feret diameter (the maximum and minimum distance between two parallel lines restricting the motor unit outline) and cross‐sectional area were computed from single‐slice acquisitions, and the shape complexity was calculated using the Hausdorff box‐counting method using a MATLAB (Mathworks version r2021a) script [33]. To produce motor unit recruitment curves, the average brightness of the voxels in the anterior compartment of a single slice was measured across all stimulation strengths.

The Hausdorff box‐counting method is a technique used to quantify the complexity of a shape by estimating its fractal dimension. This method involves overlaying a grid of boxes of a certain size over the shape and counting the number of boxes that contain part of the shape. The process is repeated with boxes of different sizes, and the relationship between the number of boxes and the size of the boxes is analyzed. This dimension provides a measure of the shape's complexity, with higher values indicating more intricate structures [34].

Motor unit diameters and shape complexity scores were measured using previously described methods [2]. Motor unit diameters and shape complexity scores were compared to healthy participants aged > 65 using the two‐sample *t* test (MATLAB *ttest2* function). These were selected from existing unpublished data collected using the same scanning protocol.

### 2.2 Structural Analysis

Dixon‐sequence scans were segmented manually using ITKsnap, version 4.20 [35] into anterior (muscles supplied by the fibular nerve including tibialis anterior and extensor digitorum longus) and posterior (muscles supplied by the tibial nerve including gastrocnemius, soleus, and tibialis posterior) compartments. Segmentation was confirmed by a clinical radiologist (L.B.). The fat‐free contractile muscle volume was calculated from Dixon sequences using ITKsnap.

Contractile muscle volume was correlated with MRC power grading for each lower limb by performing ordinal regression using Spearman's *ρ* in SPSS v29 (IBM, Armonk, NY, USA).

## 3 Results

Twelve participants were recruited based upon the inclusion criteria. Of these: one participant could not be scanned due to a flexion deformity preventing their leg safely fitting into the bore of the scanner; two showed no reliable motor unit activation at all; and one in whom data were lost prior to analysis. This left eight participants for analysis, with a mean age of 75 years (range 68–80), six of whom were female (Table 1). The mean FSS was 44.1 (27–55). Examination of lower limb MRC power rating ranged from 0 to 5 (Table 1).

**TABLE 1:** Participant characteristics.

Participant	Age (years)	Sex	Side scanned	Ankle strength (MRC)				FSS sum
				Left		Right		
				DF	PF	DF	PF	
P03	80	F	L	3	5	0	0	42
P04	78	F	L	4	4	1	1	39
P05	77	F	L	5	5	0	0	42
P06	68	F	R	0	4	2	4	52
P07	74	M	L	4	4	0	0	45
P09	76	F	L	4	4	4	3	55
P11	73	F	R	5	5	4	2	51
P12	79	M	L	2	4	5	5	27

Abbreviations: DF, dorsiflexion; FSS, fatigue severity score; MRC, Medical Research Council strength grading out of 5; PF, plantarflexion.

The motor unit data were compared to 19 adults aged 65 years or older (*n* = 6 female, mean age 73.7, range 67–82). MRC and fatigue score measurements were not available in this retrospective comparison cohort.

### 3.1 Muscle MRI Features

**FIGURE 1:**
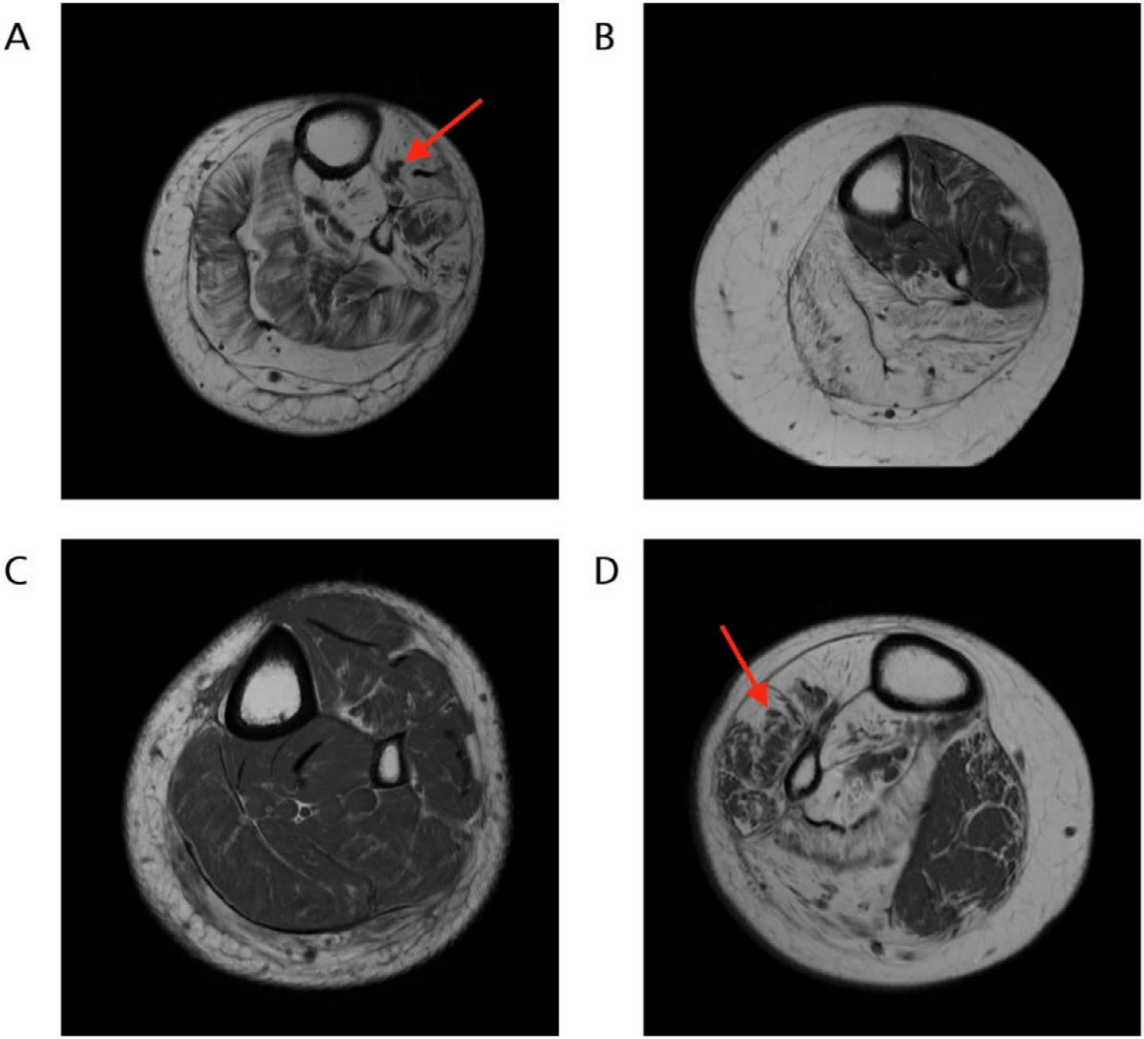
T1‐weighted muscle MRI of less‐affected legs of participants at the level of mid‐calf. (A) P03 demonstrating diffuse fatty infiltration throughout the muscle with neuromuscular “islands” of sparing in tibialis anterior (arrow). (B) P04—extensive fatty replacement in the posterior compartment with patchy fatty infiltration in the anterior compartment. (C) P05—relatively normal muscle with little fatty infiltration. (D) P06—extensive fatty infiltration and neuromuscular islands (arrow) throughout anterior compartment with relative sparing of medial head of gastrocnemius.

The structural images of the participants were heterogenous in appearance, with varying patterns of fat infiltration within, and across, different muscles of the anterior and posterior compartments (Figure 1). The degree of fatty infiltration was highly variable between and within subjects, with almost normal appearing muscles or muscle regions alongside areas of almost complete fatty infiltration.

Contractile muscle volume was also variable, with mean anterior compartment volume of 79 cm^3^, and ranging from 6.9 to 184 cm^3^. The muscle volume was moderately to strongly correlated with MRC power scores for the anterior (Spearman's *ρ* 0.828 *p* < 0.0001) and posterior compartments (Spearman's *ρ* 0.663 *p* = 0.001). Visual inspection of MRC power scores versus contractile muscle volume showed that while there was an association, there was overlap of contractile muscle volumes at different MRC power grading (Figure S1).

### 3.2 Motor Unit Characteristics

**FIGURE 2:**
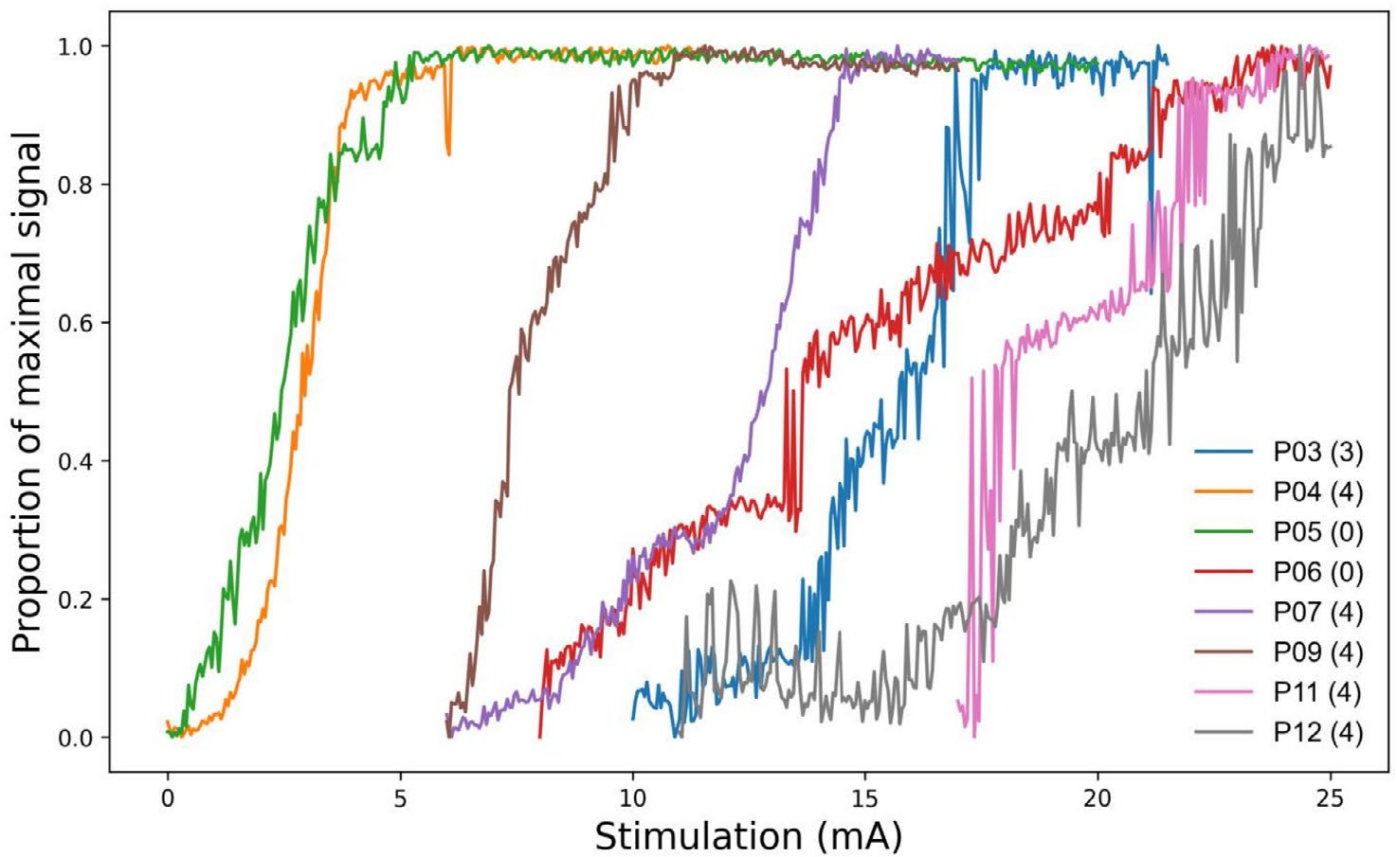
Relative pulsed gradient spin echo (PGSE) recruitment curves, demonstrating the progressive increase in signal intensity across a single slice of the segmented anterior compartment, associated with increased activation of motor units via higher stimulation currents. This shows a continuous recruitment pattern in some individuals, and a discrete recruitment with large step sizes and alternation in others. Legend indicates participant ID and the MRC force grading of ankle dorsiflexion for that limb.

The recruitment curves appeared continuous and sigmoid in three participants (P04, P05, P09) and discrete with large step increments in the remaining five (P03, P06, P07, P11, P12) (Figure 2). Alternation of single motor unit activity was seen in stimulation currents between 9.6 and 21.3 mA. Visual inspection indicated there was no clear relationship between the shape of the recruitment curve and the MRC strength scores.

**FIGURE 3:**
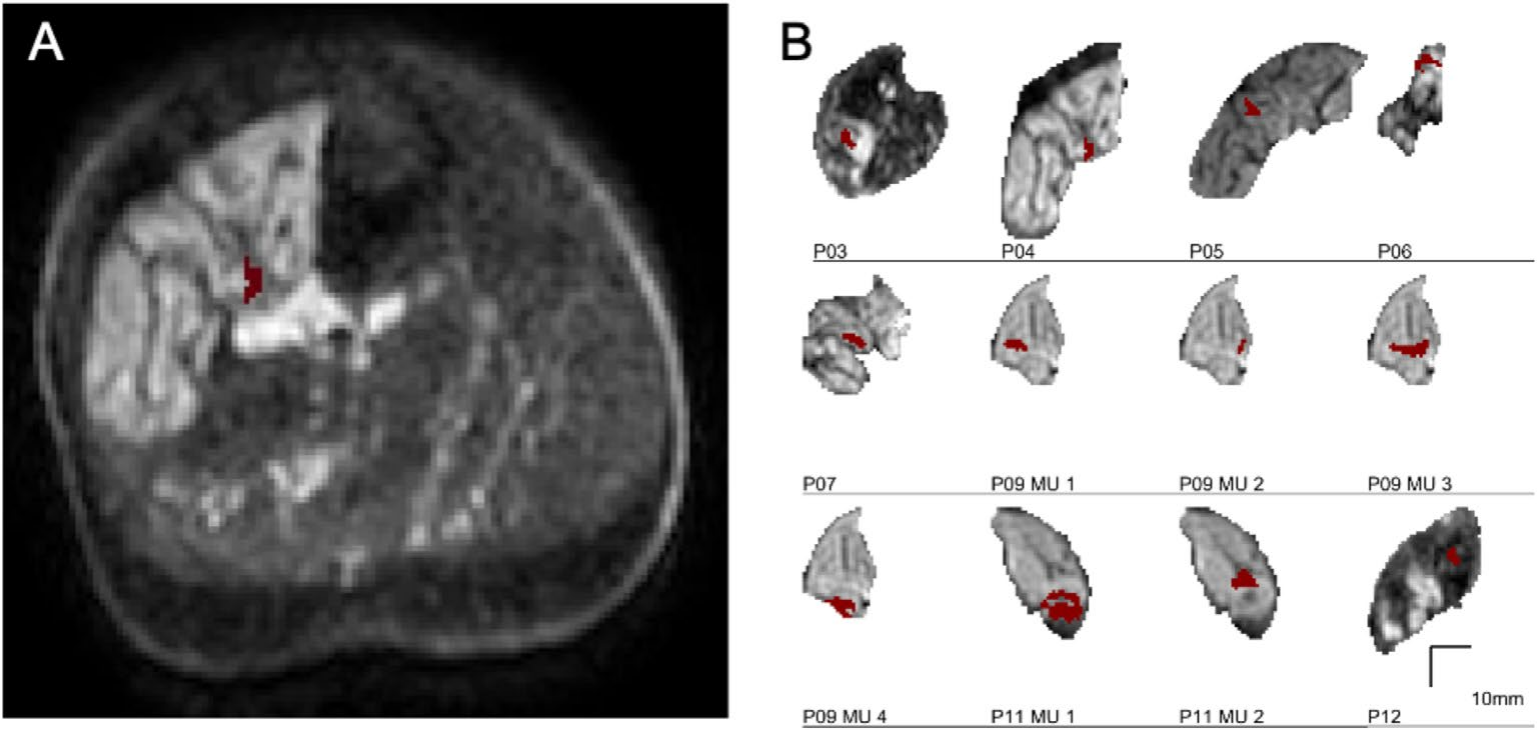
(A) Axial single‐slice motor unit profiles in the anterior compartment of the leg. (B) Each image is a single motor unit, with participant ID and motor unit number indicated.

A single motor unit was identified in the anterior compartment of six participants, with two individuals demonstrating two and four units with distinct and noncontiguous patterns of recruitment, respectively (Figure 3). In healthy controls, a single motor unit was identified for each participant.

In the single‐slice (two‐dimensional) imaging, the mean and standard deviation (SD) of the motor unit maximum Feret diameter was 10.3 ± 3.1 mm (range 7.2–16.8 mm) compared to 8.4 ± 5.2 mm (range 2.8–20.5 mm) in controls (*p* = 0.34). The mean and SD of the motor unit minimum Feret diameter was 5.5 ± 2 mm (range 2.5–10.6 mm), compared to 3.7 ± 1.9 mm (range 2–6.8 mm) in controls (*p* = 0.01). The mean and SD motor unit area was 28.2 ± 18.8 mm^2^, compared to 19.7 ± 16.2 mm^2^ in healthy controls (*p* = 0.24) (Table 2).

**TABLE 2:** Single axial slice motor unit dimensions and Hausdorff box‐counting shape complexity of polio survivors and grouped healthy controls.

Participant	MU number	Max Feret diameter (mm)	Min Feret diameter (mm)	Cross‐sectional area (mm^2^)	Hausdorff complexity
P03	1	14.2	5.3	27	0.6074
P04	1	13.0	4.2	26	0.7114
P05	1	7.8	4.9	18	0.5113
P06	1	7.2	4.9	22	0.5198
P07	1	8.9	3.9	21	0.553
P09	1	7.6	4	26	0.5407
	2	5.8	2.5	8	0.3333
	3	14.9	6	44	0.6293
	4	10.8	6.5	37	0.5879
P11	1	14.3	10.6	83	0.8301
	2	9.9	6.8	37	0.6393
P12	1	8.6	6.4	27	0.6001
Controls (mean ± SD)		8.4 ± 5.2	3.7 ± 1.9	19.7 ± 16.2	0.45 ± 0.2

Abbreviation: MU = motor unit.

The mean and SD motor unit shape complexity as computed using the Hausdorff metric was 0.59 ± 0.12 compared to 0.45 ± 0.2 in controls. There was a significant difference when comparing polio survivors to the controls (*p* = 0.03).

To further explore the relationship between the motor unit morphologies and the macroscopic tissue volume of the limb in polio survivors, we assessed the correlation between contractile muscle volume and the motor unit shape complexity measure, and no correlation was found (Spearman's *ρ* = 0.51, *p* = 0.09).

**FIGURE 4:**
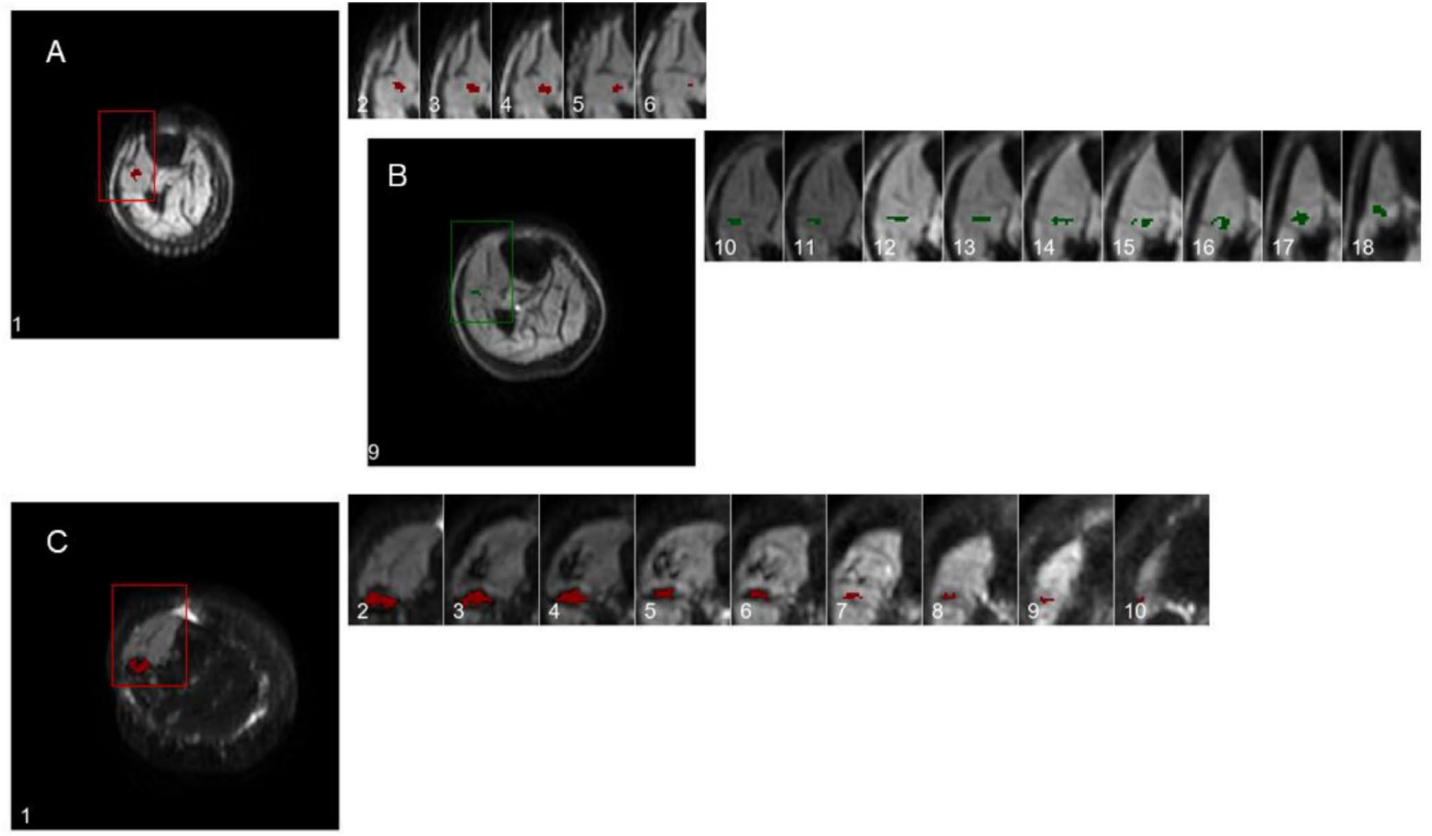
Motor unit profiles through sequential axial sections of the lower leg numbered from proximal (1) to distal (22), and zoomed in on the relevant area. (A and B) Subject P09 showing a motor unit at slices 1–6 and another at slices 9–18. (C) Subject P11 showing a single large motor unit in slices 1–10.

In the 22‐slice (three‐dimensional) imaging, six participants were excluded from analysis. Two of these showed excessive spontaneous activity at rest, one showed multiple motor units that overlapped in activation current and territory, and in three more the motor unit showed insufficient activations to produce a consistent motor unit profile between slices. Of the remaining two participants, one showed two discrete motor units while another showed a single motor unit (Figure 4). Motor units extended between 6 and 10 cm longitudinally and the maximum Feret dimensions across all slices ranged from 3.6 to 16.1 mm (Table 3).

**TABLE 3:** Multiple‐slice (three‐dimensional) measurements of motor units.

Participant	Motor unit number	Max Feret diameters, range (median)	Number of slices
P09	1	3.6–9.1 mm (7.62)	6
	2	6.7–11.2 mm (9.35)	10
P11	1	4.5–16.1 mm (11.3)	10

## 4 Discussion

Using motor unit MRI combined with in‐scanner nerve stimulation we show that the motor units in survivors of polio have a similar maximum Feret diameter and cross‐sectional area to controls, but both the minimum Feret diameter and shape complexity were significantly increased. This suggests that reinnervation occurs within the original motor unit territory, results in an increased fiber density, and that this process is not uniform across the motor unit.

The implication of this limited spatial area within which reinnervation occurs is clear. As motor units are lost in a neurogenic disease, the surviving units are able to extend collateral nerve branches to reinnervate fibers within their immediate territory, but these do not extend to reinnervate distant fibers. With limited motor unit loss there is sufficient overlap that all of the denervated fibers lie within a surviving motor unit's territory, and potentially can be reinnervated. However, with further motor unit loss, regions of muscle containing no overlapping survivors will occur, and further motor unit loss will result in permanent muscle fiber denervation and ultimately fatty replacement. This insular remodeling phenomenon may be the reason that neurogenic “islands” have been shown in a number of muscle MRI studies of neurogenic (but not myopathic) diseases [36]. We have highlighted a number of muscle MRI scans with patchy, heterogenous changes across muscle groups, including some with the same pattern of neuromuscular “islands.” Indeed one of the participants showed a single motor unit which alternated with no overlapping motor units (Figure 4C), demonstrating that all of the muscle fibers in that region belonged to a single motor unit. Despite this, the level of wasting of a muscle, representing the likely degree of polio involvement, was not correlated with measures of motor unit shape complexity.

This technique reveals an important research direction in assessing the neurogenic motor unit. Since we are able to reliably activate a motor unit and detect its location and shape within the muscle, MUMRI may be useful as a longitudinal assessment of a motor unit's health [20, 37].

We were able to obtain 3D images of only three motor units in two participants, which was a lower yield than our previous study in healthy participants [22]. Partly this was due to the difficulty in identifying discrete, non‐overlapping motor units in a given slice, and partly due to difficulties in identifying the same motor unit in adjacent slices. This may be due to the activation characteristics of neurogenic motor units, which show a higher relative spread of threshold variability compared to healthy units [38], and therefore will alternate over a wider current range than healthy units, producing fewer activations. Despite the low yield, the longitudinal extent of the imaged units is comparable to those in healthy controls (mean 8.0 ± 3.8 cm).

Our study has important limitations. While we identified our polio survivor cohort from the British Polio Fellowship and selected according to established criteria, these diagnoses are historical by 60 or more years. Polio itself is a heterogenous disease, and the intervening duration will further contribute to the heterogeneity of this population. This was seen in the variability of involvement on structural MRI sequences, showing differing degrees of fatty infiltration in different compartments, resulting in reduced contractile muscle volume which in turn was associated with reduced power rating.

There were important sex differences between our polio survivors and control cohort, with a greater proportion of female participants in the polio cohort (6/8) than controls (6/18). Important differences in muscle properties are seen between sexes, with females showing lower MUP amplitudes and higher firing rates than males [39], as well as a higher motor unit count [40], and this may have had a bearing on the differences between cohorts.

While we were able to identify single motor units in 8 out of 12 participants, a fixed‐flexion deformity prevented one individual from fitting into the bore of the scanner, and no motor unit activity could be found in two participants, potentially limiting the utility of this technique in late‐stage neurogenic diseases.

Despite longstanding efforts to eradicate the disease, polio remains prevalent in several regions of the world, most notably Pakistan and Afghanistan. With increasing global migration and vaccine hesitancy, the number of polio cases is likely to rise [41]. While our study population was exclusively UK patients who had contracted the disease during the polio outbreaks in the 1950s and 60s, a new group of far younger survivors of polio is being recognized who also need accurate diagnosis and monitoring. We believe that MUMRI may have a role alongside conventional EMG in achieving this.

## Author Contributions

**Stuart Maitland:** conceptualization, investigation, writing – original draft, methodology, writing – review and editing, software, formal analysis, project administration, and data curation. **Matthew Birkbeck:** conceptualization, methodology, writing – review and editing, formal analysis, and data curation. **Ian Schofield:** conceptualization, investigation, methodology, writing – review and editing, formal analysis, project administration. **Lawrence Best:** formal analysis, writing – review and editing. **James Scott:** writing – review and editing, formal analysis. **Andrew Blamire:** conceptualization, investigation, funding acquisition, writing – review and editing, supervision. **Roger G. Whittaker:** conceptualization, investigation, funding acquisition, writing – review and editing, formal analysis, project administration, supervision, and resources.

## Funding

This work was funded by the NIHR Newcastle Biomedical Research Centre. The NIHR Newcastle Biomedical Research Centre is a partnership between Newcastle University and Newcastle Hospitals, funded by the National Institute for Health and Care Research (NIHR). This work presents independent research funded and supported by the NIHR Newcastle Biomedical Research Centre. The views expressed are those of the author(s) and not necessarily those of the NIHR or the Department of Health and Social Care.

## Ethics Statement

We confirm that we have read the Journal's position on issues involved in ethical publication and affirm that this report is consistent with those guidelines. Ethical approval was obtained from the Newcastle University Faculty of Medical Sciences Ethics Committee (references 2603/34701 and 1852/525/2020).

## Consent

All participants were provided with information and materials prior to recruitment and freely provided consent to take part in the study.

## Conflicts of Interest

The authors declare no conflicts of interest.

## Supporting information


**Figure S1:** Fat‐free contractile muscle volume versus MRC power.

**Table S1:** Scan parameters for various sequences.

## Data Availability

The data that support the findings of this study are available on request from the corresponding author. The data are not publicly available due to privacy or ethical restrictions.
