# Supplementary material for: MRI of Neurogenic Human Motor Units Following Poliomyelitis
Source: Muscle Nerve. 2025 Dec 14;73(3):403–11. doi: 10.1002/mus.70107 (PMC12888830; doi:10.1002/mus.70107)
Supplement: Supplementary file 1 — Figure S1: Fat‐free contractile muscle volume versus MRC power. [file MUS-73-403-s002.pdf]

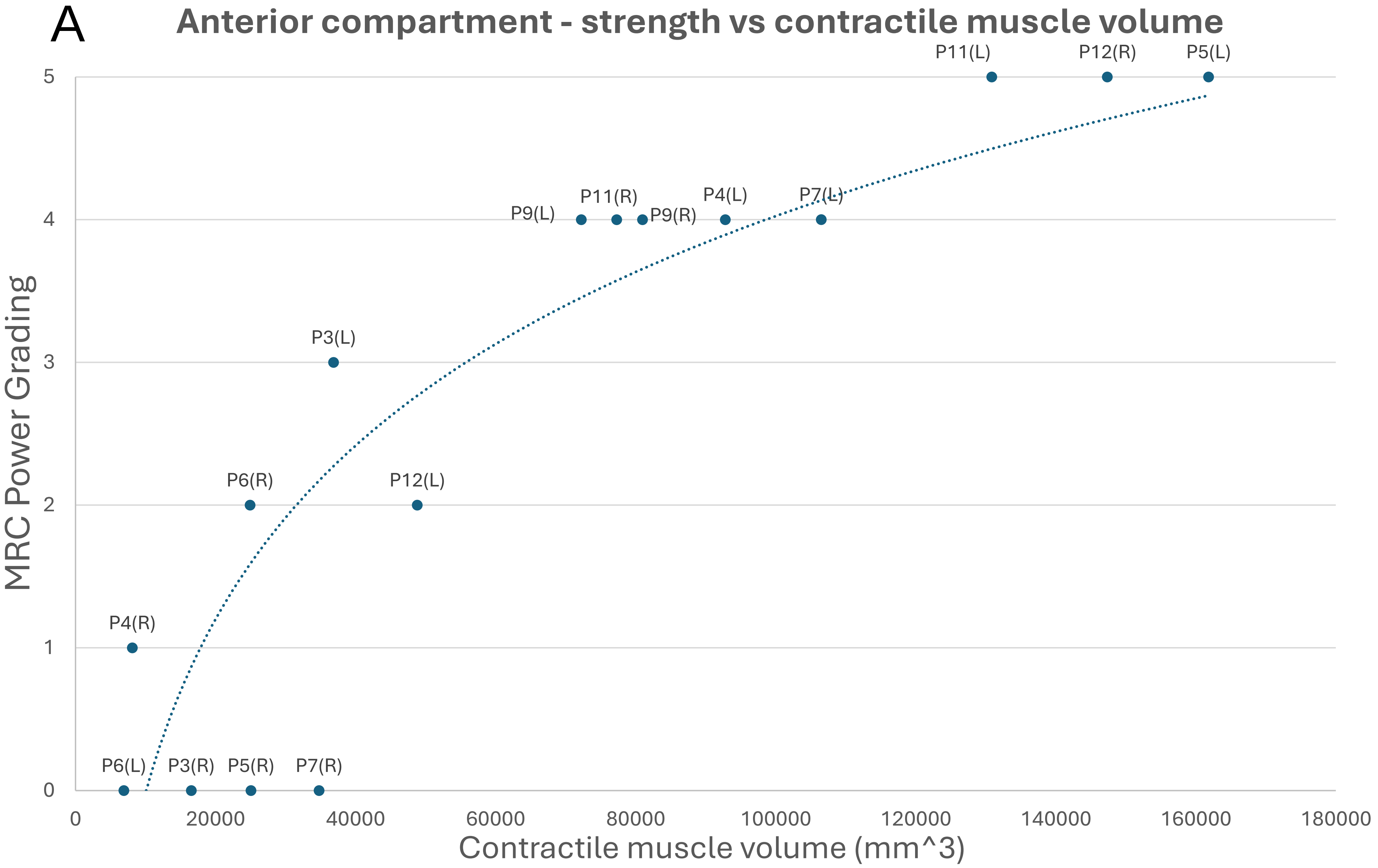

A
Anterior compartment - strength vs contractile muscle volume
MRC Power Grading
Contractile muscle volume (mm^3)
0
1
2
3
4
5
0
20000
40000
60000
80000
100000
120000
140000
160000
180000
P11(L)
P12(R)
P5(L)
P9(L)
P11(R)
P9(R)
P4(L)
P7(L)
P3(L)
P6(R)
P12(L)
P4(R)
P6(L)
P3(R)
P5(R)
P7(R)

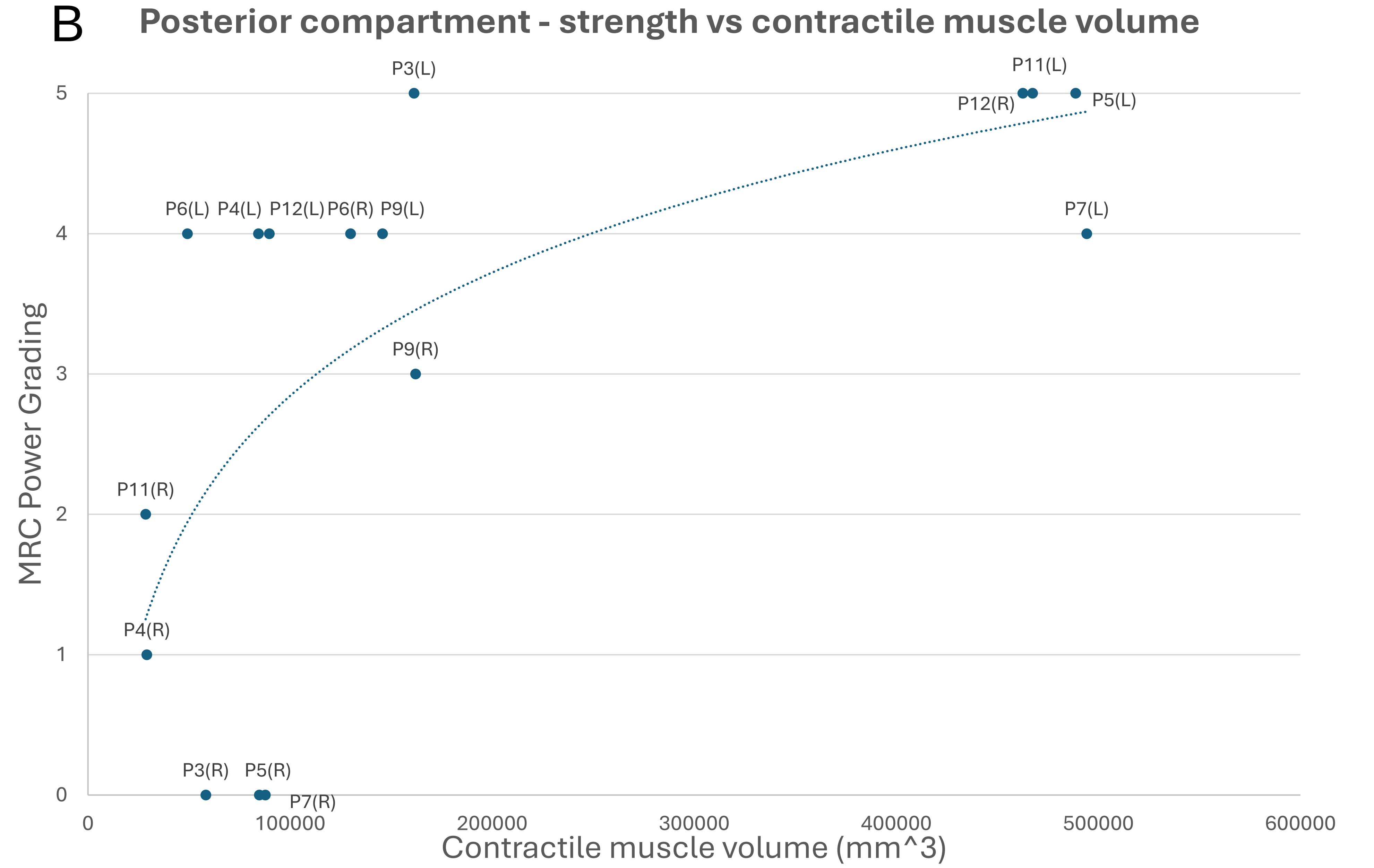

B
Posterior compartment - strength vs contractile muscle volume
MRC Power Grading
Contractile muscle volume (mm^3)
0
1
2
3
4
5
0
100000
200000
300000
400000
500000
600000
P3(L)
P11(L)
P12(R)
P5(L)
P6(L)
P4(L)
P12(L)
P6(R)
P9(L)
P7(L)
P9(R)
P11(R)
P4(R)
P3(R)
P5(R)
P7(R)
