# Supplementary material for: MRI of Neurogenic Human Motor Units Following Poliomyelitis
Source: Muscle Nerve. 2025 Dec 14;73(3):403–11. doi: 10.1002/mus.70107 (PMC12888830; doi:10.1002/mus.70107)
Supplement: Supplementary file 2 — Table S1: Scan parameters for various sequences. [file MUS-73-403-s001.pdf]

Scan	Field of View (AP,RL,FH) (mm)	Voxel Size (mm)	No. Slices; Slice Gap (mm)	TE (ΔTE)/TR (ms)	Averages	Flip Angle °	No. Dynamics	Diffusion Gradient (Δ/δ) (ms)	Acceleration	Fat Suppression	Acquisition Time (mm:ss)
T1 TSE	160 x 160 x 300	0.417 x 0.5 x 10	30;0	20/633	1	90	-	-	-	-	03:29
Dixon	384 x 168 x 100	1 x 1 x 10	4;20	3.45 (1.15)/180	3	8	-	-	-	-	04:33
PGSE Fasciculation	384 x 168 x 100	2.5 x 2.5 x 10	4;20	33/1000	1	90	100	15.8/6.5	SENSE = 2	SPAIR, SSGR, Olefinic Pre-Pulse	01:43
PGSE Set Up	160 x 160 x 40	1.5 x 1.5 x 10	2;20	36/1000	1	90	20	17.1/2.2	SENSE = 2	SPAIR, SSGR, Olefinic Pre-Pulse	00:20
PGSE Single Motor Unit	160 x 160 x 40	1.5 x 1.5 x 10	2;20	36/1000	1	90	360	17.1/2.2	SENSE = 2	SPAIR, SSGR, Olefinic Pre-Pulse	06:03
PGSE 3D Scan	160 x 160 x 120	1.5 x 1.5 x 10	12;0	37/6000	1	90	125	17.3/2.2	SENSE = 2	SPAIR, SSGR, Olefinic Pre-Pulse	12:30
